# Small‐Molecule Neuromedin U Receptor 2 Agonists Suppress Food Intake and Decrease Visceral Fat in Animal Models

**DOI:** 10.1002/prp2.425

**Published:** 2018-08-23

**Authors:** Catherine M. Sampson, James M. Kasper, Daniel E. Felsing, Sweta R. Raval, Na Ye, Pingyuan Wang, Igor Patrikeev, Erik Rytting, Jia Zhou, John A. Allen, Jonathan D. Hommel

**Affiliations:** ^1^ Center for Addiction Research Department of Pharmacology and Toxicology University of Texas Medical Branch Galveston TX USA; ^2^ College of Pharmaceutical Sciences Soochow University Suzhou Jiangsu China; ^3^ Center for Biomedical Engineering University of Texas Medical Branch Galveston TX USA; ^4^ Department of Obstetrics and Gynecology University of Texas Medical Branch Galveston TX USA

**Keywords:** calcium, cyclic AMP, feeding, high‐fat diet, Neuromedin U receptor 2, obesity, small‐molecule agonist

## Abstract

Obesity is a growing public health concern, with 37.5% of the adult population in need of therapeutics that are more efficacious with a better side effect profile. An innovative target in this regard is neuromedin U, a neuropeptide shown to suppress food intake and attenuate weight gain in animal models. These effects of neuromedin U on feeding behavior are thought to be related to agonism at the centrally expressed neuromedin U receptor 2 (NMUR2). As peptides present unique challenges that limit their therapeutic potential, the discovery of small‐molecule NMUR2 agonists is needed to validate the targets therapeutic value, but to date, none have been evaluated in any animal model of disease. We therefore assessed two small‐molecule NMUR2 agonists for their in vitro signaling and their in vivo efficacy. The NMUR2 agonists were synthesized and both NMUR2 agonists, NY0116 and NY0128, decreased cAMP while stimulating calcium signaling in stably expressing NMUR2 HEK293 cells. When small‐molecule NMUR2 agonists were tested in vivo, acute administration significantly decreased high‐fat diet consumption. Repeated administration of the compounds decreased body weight and more specifically, decreased the percentage of visceral adipose tissue (VAT) in obese mice. These results have confirmed small‐molecule NMUR2 agonists are efficacious in animal models to decrease fat content, food intake, and body weight, suggesting NMUR2 is a promising therapeutic target for metabolic disorders.

**Abbreviations**
DMEM: Dulbecco's modified Eagle's medium
DIO: diet‐induced obese
FBS: Fetal Bovine Serum
HBSS: Hanks balanced salt solution
LCPS: luminescence light counts per second
micro‐CT: microcomputed tomography
NMS: neuropeptide neuromedin S
NMU: Neuromedin U
NMUR1/2: NMU receptor ½
VAT: visceral adipose tissue

## 1 INTRODUCTION

Obesity remains a troubling health problem that demands a more mechanistic appreciation of the molecular and neural basis of food consumption.^1^ The complex nature of obesity, a disorder partially characterized by overconsumption of energy‐dense foods, creates challenges for developing therapeutics.^2^ Although 80 million people in the United States are obese,^3^ the available medications and surgical interventions suffer from modest efficacy and/or numerous side effects.^4^ Our objective is to identify and validate new druggable targets via pharmacological approaches that exploit an enhanced understanding of mechanisms underlying feeding behavior. Neuromedin U (NMU) is a neuropeptide, synthesized in the lateral hypothalamus and expressed in all mammals.^5^ NMU‐deficient mice are hyperphagic, obese, and have decreased metabolic activity.^6^ Conversely, systemic administration of NMU peptide suppresses food intake^7^ and causes weight loss.^8^ NMU gene variants have also been associated with obesity,^9^ whereas studies have also shown NMU to be upregulated in calorie restricted animals.^10^ Together, these data suggest that NMU plays an important role in regulation of feeding and body weight.

To leverage the desirable antiobesity effects of NMU, it is important to understand the primary sites of action for NMU. There are two high‐affinity receptors for NMU, neuromedin U receptor 1 (NMUR1) and NMUR2. These receptors also bind the endogenous neuropeptide neuromedin S (NMS). NMUR1 is enriched in the periphery, and may be involved in regulation of the immune system and responses to pain.^11^, ^12^ NMUR2, however, is enriched in the brain, specifically the hypothalamus, confirmed by qRT‐PCR and mRNA analyses.^13^, ^14^, ^15^ NMUR2 is highly expressed in regions associated with food reward 16 and mediates the central effects of NMU (and NMS) on food intake and body weight.^8^, ^17^

NMUR2 may represent a druggable target and a potential access point for the control of important neural pathways underlying food intake and ultimately obesity. NMUR2 is a G‐protein‐coupled receptor, suggested to couple to both G_i/o_ and G_q_ and was de‐orphaned over 10 years ago.^7^, ^18^ Following the discovery that NMUR2 (aka FM‐4, TGR‐1) is stimulated by NMU, NMUR2 was shown to play an important role in regulating food intake and body weight. Knockout of NMUR2 in mice causes hyperphagia, weight gain, and decreased metabolic activity.^17^ Although not all studies observed the same effect on food intake after NMUR2 knockout.^8^ Furthermore, our laboratory discovered that NMUR2 regulates specific types of feeding behavior in animal models. That is, knockdown of NMUR2 in the paraventricular nucleus of the hypothalamus potentiates binge‐type eating, increases consumption of a high‐fat diet, and stimulates weight gain.^19^ Recent studies have also shown that NMU administered to the paraventricular nucleus of the hypothalamus also decreases intake^20^ and motivation for high‐fat diet.^5^ Taken together, NMUR2 activation shows therapeutic promise for treating obesity.

**Figure 1:**
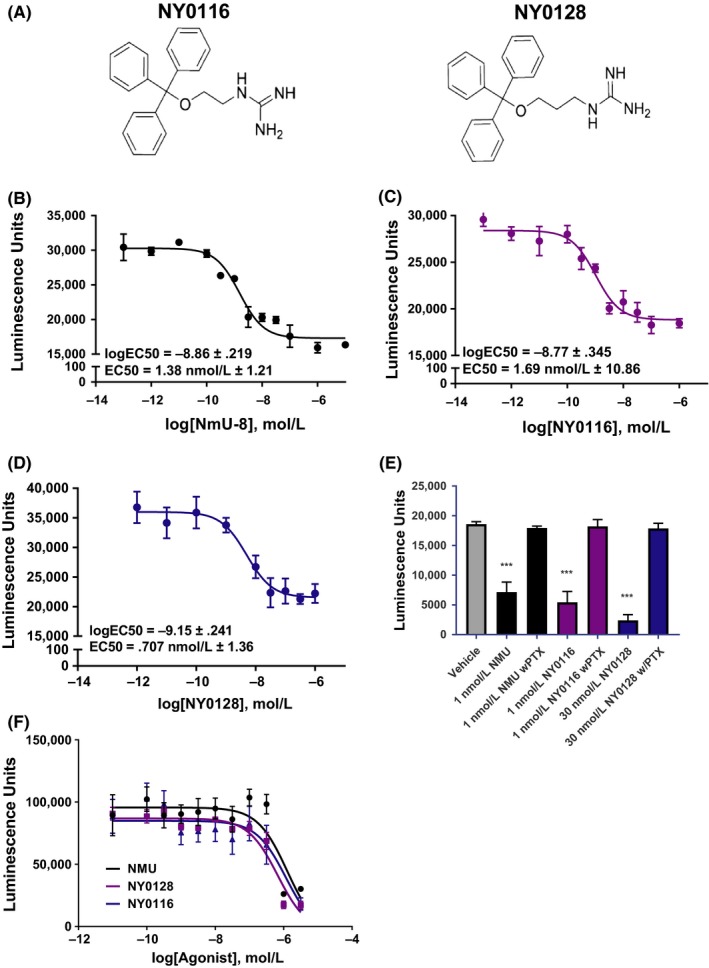
Pharmacological characterization of NMUR2 agonists NY0116 and NY0128 in Gi mode cAMP assays. (A) The chemical structures of NY0116 and NY0128. (B) NMU‐8 dose dependently decreases 1 nmol/L isoproterenol‐induced cAMP levels in HEK293 cells stably expressing NMUR2. (C) NY0116 decreases cAMP in HEK293 cells stably expressing NMUR2. (D) NY0128 also dose dependently decreased cAMP levels in HEK293 cells stably expressing NMUR2. (E) PTX pretreatment of cells prevented NMU‐8, NY016, or NY0128‐mediated decreases of cAMP, indicating agonist dependence for Gi/o coupling to the NMUR2 to decrease cAMP. (F) cAMP levels in HEK293 cells not expressing NMUR2 were not altered by administration of compounds at concentrations seen to inhibit cAMP in HEK293 stably expressing cells, indicating the decrease in cAMP seen in the assays depicted in Figure 1B–D is due to activation of NMUR2. Means ± SEM. for 15‐16 independent experiments (*n *=* *15–16); ***P <* *0.001 vs control, one‐way anova with Dunnett's posttest. All dose‐response curves are representative plots, performed in triplicate. NMUR1/2, NMU receptor ½

Several small‐molecule agonists for NMUR2 were discovered by Meng et al.^21^ via a high‐throughput screen. A series of the original compounds were investigated in live‐cell assays, allowing a preliminary structure‐activity relationship. In particular, two structurally similar compounds, NY0116 and NY0128 (chemical structures shown in Figure 1A) containing a lipophilic trityl motif and a hydrophilic guanidine scaffold were demonstrated as agonists against human NMUR1 (hNMUR1) and human NMUR2 (hNMUR2) via intracellular calcium mobilization assays.^21^ In this previous work, NY0116 had EC_50_ values of 27.76 μmol/L for hNMUR1 and 13.61 μmol/L for hNMUR2, whereas NY0128 had EC_50_ values of 29.99 μmol/L for hNMUR1 and 10.30 μmol/L for hNMUR2.^21^ However, the potential of these small‐molecule agonists to suppress food intake or alter body composition in animals has not been evaluated, creating uncertainty about the potential of NMUR2 as a target for obesity. Here we have filled a major gap in our understanding of NMUR2 as a therapeutic target by performing the first test of small‐molecule NMUR2 agonists in any animal model of disease. These results support NMUR2 as a druggable target that can suppress food intake and improve body composition.

## 2 MATERIALS AND METHODS

### 2.1 Ethics statement

All experiments were carried out in accordance with all national and local guidelines and regulations, with the Guide for the Care and Use of Laboratory Animals 27, and with the approval of the Institutional Animal Care and Use Committee at The University of Texas Medical Branch. All efforts were made to minimize animal suffering and to reduce the number of animals used.

### 2.2 Compounds

NY0116 and NY0128 were synthesized in‐house following a reported synthetic route 21 with optimized procedures. The chemical structures were fully characterized using H1 and C13 NMR and mass spectrometry techniques, and compound purity was verified using HPLC analysis.

### 2.3 Cell culture

Human Embryonic Kidney 293 (HEK293) cells and HEK293 stably expressing the human NMUR2 receptor (C11 cells) were obtained from GenScript (cat no. M00244 Piscataway, NJ) and a single colony expressing NMUR2 was selected for receptor signaling experiments. The HEK293 cells stably expressing a tTA‐dependent luciferase reporter gene and beta arrestin2‐TEV fusion protein (HTLA cells), were a generous gift from Dr. Bryan Roth, UNC Chapel Hill.^22^ All cells were cultured in a humidified incubator at 37°C in 5% CO_2_, up to passage 25, in complete media, containing Dulbecco's modified Eagle's medium (DMEM) (cat no. 31053028, Gibco, Carlsbad, CA), supplemented with 10% Fetal Bovine Serum (FBS) (cat no. 16000044, Gibco, Carlsbad, CA), 100 U/mL penicillin and 100 μg/mL streptomycin (cat no. BP295950; Fisher BioReagents, Pittsburg, PA). C11 NMUR2 expressing cells were cultured under stable selection conditions in complete media supplemented with 200 μg/mL zeocin (cat no. R25001; Gibco, Carlsbad, CA), whereas HTLA cells were maintained under stable selection in complete media supplemented with 2 μg/mL puromycin (cat no. A1113803, Gibco, Carlsbad, CA) and 100 μg/mL hygromycin B (cat no. 10687010; Thermo Fisher Scientific, Waltham, MA).

### 2.4 GloSensor cAMP assay

C11 cells stably expressing the NMUR2 were seeded at 1 000 000 cells/well into poly‐L‐lysine coated 6 well plates. The next day, the cells were transfected with 1 μg/well of GloSensor 22F plasmid using Lipofectamine 2000 and incubated at 37°C with 5% CO2 overnight. Cells were then seeded at a density of 50 000 cells/well into poly‐L‐lysine coated white walled, clear bottom 96 well plates and grown overnight. The next day, complete media was aspirated and replaced with 90 μL of GloSensor cAMP reagent, diluted 1:100 in 1X HBSS+20 mmol/L HEPEs and incubated at room temperature in the dark for 2 hours. After the 2 hours serum starved incubation, serial dilutions, 10 pmol/L to 10 μmol/L, of NMU‐8 and each compound were prepared and 10 μL of 10x drug was added to each well and placed back in the dark to incubate for 10 minutes. At the end of the 10 minute incubation, 11 μL of 10 nmol/L isoproterenol (final concentration of 1 nmol/L) was added to the cells to stimulate cAMP levels. After another 10 minute incubation in the dark, plates were placed in a MicroBeta^2^ (Perkin Elmer, Waltham, MA) luminescence imaging plate reader. Luminescence light counts per second (LCPS) of the GloSensor in each well, which is proportional to the concentration of cellular cAMP was determined. Data from 15 to 16 independent experiments (*n *=* *15–16), conducted in sextuplicate, are presented and is reported as light counts per second (lcps).

### 2.5 FLIPR calcium assay

Wildtype HEK293 or C11 cells stably expressing the NMUR2 were seeded at 60 000 cells/well into poly‐L‐lysine (cat no. NC0818114; Sigma Aldrich, Waltham, MA) coated 96 well black clear bottomed cell culture plates (cat no. 07200565, Thermo Fisher Scientific, Waltham, MA) and cultured in complete DMEM with 10% dialyzed FBS. The next day complete media was aspirated and replaced with 50 μL of Hanks balanced salt solution (HBSS) (cat no. 14175095; Gibco, Carlsbad, CA) and cells serum starved for 1 hour in an incubator. 50 μL of 2x FLIPR Calcium 5 dye (cat no. NC9897124, Molecular Devices, Sunnyvale, CA) with 5.0 mmol/L probenecid (final in well concentration of 2.5 mmol/L) was added to the cells and placed back into an incubator for 1 hour. Serial dilutions, 1 nmol/L to 100 μmol/L, of each compound were prepared at 5X final concentration and transferred to a 96 well source plate. Cell and drug plates were placed in a fluorescence imaging plate reader (FLIPR^TETRA^) (Molecular Devices, Sunnyvale, CA). The FLIPR^TETRA^ was programmed to read baseline dye fluorescence for 10s followed by addition of 20 μL (5x) drug/well and read for an additional 120s (acquisition 1 time/s). The maximum (peak) fluorescence observed in each well during the first 40 seconds after compound addition was determined using the FLIPR^TETRA^ ScreenWorks 4.0 program and results normalized to the average of the baseline fluorescence in each well (first 10 reads). Data from independent experiments (*n *=* *4), conducted in quadruplicate, are presented.

### 2.6 Tango beta‐arrestin activity assay

The tango beta‐arrestin assay was performed according to previously published methods^22^ with slight modification. In brief, 24 hours before transfection, 6 × 10^5^ HTLA cells were plated in a single well of a 6 well plate (cat no. 0720080, Corning, Oneonta, NY) to achieve 60%–70% confluency the following day. 24 hours after initial seeding, each well of HTLA cells were transfected with 1 μg of human NMUR2 Tango plasmid (Addgene, Cambridge, MA) using 10 μL Lipofectamine 2000 (cat no. 11668019; Invitrogen, Waltham, MA), 300 μL of OptiMEM (cat no. 31985070; Gibco, Carlsbad, CA) and 1.5 mL HTLA media. 24 hours post transfection, HTLA cells were seeded at 50 000 cells/well into poly‐L‐lysine (cat no. NC0818114; Sigma Aldrich, Waltham, MA) coated 96 well white clear bottomed cell culture plates (cat no. 07000167; Greiner Bio‐One, Monroe, NC). The following day, a 10X concentration of NMS (cat no. 3648, Tocris, Bristol, UK), NY0116 and NY0128, were prepared in the assay buffer (1X HBSS, pH 7.4 and 20 mmol/L HEPES) and added to each well. 18 hours after compound additions, media was aspirated and the cells were incubated in 50 μL/well Bright‐Glo reagent (cat no. PRE2610; Promega, Madison, WI), diluted to 20‐fold in the assay buffer, for 20 minutes at room temperature. LCPS were immediately measured using a Microbeta^2^ microplate reader (Perkin Elmer, Waltham, MA). Data from independent experiments (*n *=* *3), conducted in quadruplicate, are presented.

### 2.7 Collection of rat plasma and brain samples

Male Sprague‐Dawley rats (*n *=* *30) (Harlan Inc., Houston, TX) were single‐housed at 21.67°C and 30%‐50% relative humidity with a 12‐hour light‐dark cycle (lights on 6:00 am‐6:00 pm) and were allowed to habituate for 7 days prior to experimentation. At 8:30 am, rats were given a 30 mg/kg subcutaneous injection of either NY0116 or NY0128. At each time point, rats were anesthetized with vaporized isoflurane (VetEquip, Pleasanton, CA) and decapitated. For NY0116, three rats were sacrificed at 1.5, 4, 10, 24, and 48 hours postinjection. For NY0128, three rats were sacrificed at 45 minutes, 1.5, 4, 8, and 24 hours. At the time of death, 250 μL of blood was collected into EDTA coated tubes and the whole brain extracted for each animal. The blood samples were centrifuged at 1500 g for 10 minutes at 4°C to isolate the plasma. The plasma was transferred into sample tubes, frozen on dry ice, and stored at −80°C. The brains were rinsed well with sterile PBS, frozen on dry ice, weighed, and stored at −80°C. To homogenize the brain tissue, four volumes of 20% methanol were added to each brain sample and then processed for one minute using a rotor homogenizer (Biospec Products, Bartlesville, OK). The brain homogenates were transferred to sample tubes, frozen on dry ice, and stored at −80°C. Plasma and brain homogenate samples were shipped on dry ice to Alliance Pharma (Malvern, PA) for analysis.

### 2.8 24‐hour food intake

Male Sprague‐Dawley rats (Harlan Laboratories Inc., Houston, TX) were used, weighing 225–250g at the start of the experiments. The rats were single‐ housed in colony rooms maintained at 21.67°C and 30%–50% relative humidity with a 12‐hour light‐dark cycle (lights on 6:00 am–6:00 pm) and allowed to habituate for 7 days. Rats were fed a standard (lean) diet (*n *=* *40) (Teklad Mouse/Rat Diet 7912; Harlan Laboratories Inc., Houston, TX) or high‐fat diet (*n *=* *34) (45% kcal from fat; Open Source Diets formula D12451; Research Diets Inc., New Brunswick, NJ, USA) ad libitum, handled daily, and weighed every 3 days with a dynamic weighing apparatus (Ohaus Corp, Parsippany, NJ). To monitor intake, food hoppers were weighed every other day for 7 days. Two days before compound administration, rats were given a subcutaneous 2 mL/kg saline injection 1 hour before the onset of the dark cycle, which was repeated the next day. On the seventh day, food hoppers were removed and weighed before the rats received a subcutaneous injection of saline, NY0116, or NY0128 1 hour before the onset of the dark cycle. Hoppers were weighed 2, 4, 8, and 24 hours postinjection.

### 2.9 Repeated compound administration

64 male diet induced obese (DIO) C57BL/6J mice age 18 weeks and 8 age matched C57BL/6 control mice (The Jackson Laboratories, Bar Harbor, ME) were single‐housed as described above and allowed to habituate for 14 days prior to experimentation. DIO mice were maintained on a high‐fat diet (cat no. D12451, 45% energy from fat; Open Source Diets formula D12451; Research Diets Inc., New Brunswick, NJ, USA) and control mice were maintained on a standard brown chow rodent diet, with access to food and water ad libitum. DIO mice have been maintained on a high‐fat diet of at least 45% per kcal since weaning. 60 DIO mice were divided into seven treatment groups with one saline control group (*n *=* *12), 3 NY0116 dose groups (3, 10, 30 mg/kg; *n *=* *8/group), and 3 NY0128 dose groups (3, 10, 30 mg/kg; *n *=* *8/group). Forty‐eight hours prior to the start of the experiment, mice were given daily saline injections to allow them to acclimate to injections. Mice were then randomly divided into five groups (*n *=* *12) that consisted of animals from each treatment group, with the first group beginning treatment on a Sunday and the fifth group beginning treatment on a Thursday, with all groups ending 14 days later on their respective days of the week. Reasoning for the varied start is due to the time limitations of the micro‐CT and the number of animals that can be scanned per day. Daily doses began at 3 pm Central Standard Time (CST) and ended before the dark cycle began at 6 pm CST. Food intake, injections, and weight loss were recorded following a repeated measures design. Injection volumes were determined using the weight recorded 1 day prior to the start of treatment and re‐evaluated and adjusted after day 7 treatment administration.

### 2.10 Micro‐CT scan

Mouse whole‐body scans were performed using a Siemens Inveon MultiModality micro‐computed tomography system (Siemens Medical Solutions USA, Knoxville, TN). Briefly, mice were imaged in a prone position on the scanner radiolucent bed. Anesthesia was provided with 2% isoflurane carried by oxygen through a nose cone during the scan. The high‐resolution micro‐CT exposure settings were 70 kV voltage, 500 uA current, and 520 steps at 360°‐rotation. No contrast agent was used. To compare the amounts of visceral adipose tissue (VAT) between the groups we examined the cross section at the L5 vertebrae (Figure 5C), using Inveon Research Workplace for quantification. Total area of cross section and the areas of observed VAT inside the cross section were measured. Percentage of VAT was calculated as a ratio of VAT area to the total area of cross section expressed. The segmentation of the adipose tissue was based on natural contrast between VAT and surrounding tissues. VAT deposits were segmented out in semiautomated selection mode.^23^

### 2.11 Serum collection

Post micro‐CT scan, mice were exposed to CO_2_ and decapitated. Following decapitation, 500 μL of trunk blood was collected and allowed to incubate at room temp for >15 minutes. Following incubation, the blood samples were then centrifuged at 1500 g for 15 minutes. Following centrifugation, plasma was removed from the sample and the serum was stored at −80°C until further analysis. Serum analysis was conducted by the Texas A&M Veterinary Medicine Diagnostic Laboratory. Cholesterol, creatinine, blood urea nitrogen, aspartate aminotransferase, and alanine aminotransferase and glucose levels were analyzed in the serum. Due to hemolysis of samples, some samples were unable to be accurately analyzed and were removed from further analysis.

### 2.12 Data analysis and statistics

All data were analyzed using GraphPad Prism 7.0 software (La Jolla, CA). Data from calcium flux and beta‐arrestin recruitment are presented as half maximum (EC_50_) and maximum (E_MAX_) values, representing potency and efficacy, as computed by GraphPad using a four parameter nonlinear regression curve‐fitting algorithm. A one‐way anova with Dunnett's post hoc test was used to compare statistical differences in the results. Data from in vivo studies were analyzed using one or two‐way anova with Bonferroni post hoc test.

## 3 RESULTS

### 3.1 NMUR2 agonists NY0116 and NY0128 potently inhibit cAMP

The pharmacological activity of NMUR2 agonists NY0116 and NY0128 (Figure 1A) were evaluated in a G_i_ mode cAMP assay using HEK293 cells stably expressing hNMUR2. Cells were stimulated with 1 nmol/L isoproterenol to elevate cAMP levels, which is required to see an inhibition of cAMP via G_i_ ‐coupled GPCRs.^24^ Full dose responses (1 nmol/L to 100 μmol/L) of NMU‐8, NY0116, and NY0128 were then tested to assess their activity to signal via NMUR2 and G_i_‐mediated signaling. NMU‐8 potently inhibited isoproterenol‐stimulated cAMP in a dose‐dependent manner (Figure 1B), with low nanomolar EC_50_. Similarly, potent dose‐dependent inhibition of cAMP was observed when testing the NMUR2 agonists NY0116 and NY0128. EC50 values estimated from regression curves were 1.38 nmol/L ± 1.21 for NMU‐8, 1.69 ± 10.96 nmol/L for NY0116 (Figure 1C) and 0.71 ± 1.36 nmol/L for NY0128 (Figure 1D) (Means ± SEM, *n *=* *15–16 independent experiments) (Table 1). To validate the inhibition of cAMP by these NMUR2 agonists was G_i_ ‐mediated, the stable NMUR2 expressing cells were pretreated with 150 ng/mL pertussis toxin for 18 hours to uncouple G_i_ proteins. Pertussis toxin entirely reversed the observed cAMP inhibition, indicating the potent agonism of these ligands requires NMUR2‐G_i_ coupling (Figure 1E). To further insure the inhibition was due to activation of NMUR2, cells not expressing NMUR2 were also evaluated (Figure 1F). There was no effect on cAMP at concentrations of compounds that decreased cAMP in the NMUR2 stably expressing cells.

**Table 1:** It shows the EC50 and Emax (relative to NmU‐8) values of NmU‐8, NY0116, and NY0128 when evaluated in Gi mode

Treatment	G_i/o_ EC_50_ (nmol/L)	G_i/o_ E_max_ (% NmU‐8)
NmU‐8	1.38 nmol/L ± 1.21	–
NY0116	1.69 nmol/L ± 10.86	139.3% ± 36.09%
NY0128	0.71 nmol/L ± 1.36	189.5% ± 34.94%

NMU, Neuromedin U.

### 3.2 NMUR2 agonists NY0116 and NY0128 mobilize calcium

**Figure 2:**
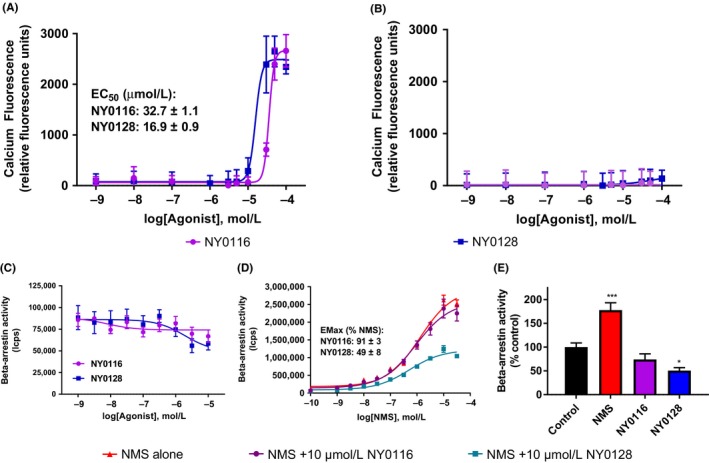
Pharmacological characterization of NMUR2 agonists NY0116 and NY0128 in calcium and Beta‐arrestin assays. (A) NY0116 and NY0128 activate calcium release in HEK293 cells stably expressing the human NMUR2. (B) Neither agonist increases calcium in wild‐type HEK293 cells that lack expression of NMUR2. [C]) NMS, but not NY0116 or NY0128, dose dependently activates beta‐arrestin recruitment in the Tango assay. (E) 1 nmol/L treatment of cells with the native hormone neuromedin‐S (NMS) activated, whereas 10 μmol/L NY0128 significantly decreased basal NMUR2‐mediated beta‐arrestin activity (*P <* *0.05 vs. control, *n *=* *3, one‐way anova). All dose‐response curves are representative plots, performed in quadruplicate, with similar results observed in 3 to 4 independent experiments. NMUR1/2, NMU receptor ½

To further assess the pharmacology of NY0116 and NY0128, full dose responses (1 nmol/L to 100 μmol/L) of ligands were tested in calcium mobilization assays by assessing calcium dye fluorescence in living cells (Figure 2A). Robust responses to both ligands were observed indicating G_q_‐mediated NMUR2 signaling. EC_50_ values estimated from regression curves were 32.7 ± 1.1 μmol/L for NY0116 and 16.9 ± 0.9 μmol/L for NY0128 (Means ± SEM, *n *=* *4). Although the observed potencies for both ligands were low, they are similar to the micromolar potencies previously reported for NMUR2 calcium signaling.^21^ These calcium mobilization effects were not due to off‐target compound activity as no significant increase in calcium mobilization was observed in wild‐type HEK293 cells lacking the NMUR2 (Figure 2B). In addition, vehicle treatment, with up to 0.5% DMSO (DMSO concentration at 100 μmol/L drug point), showed no effect on calcium mobilization in either NMUR2 stable or wild‐type HEK293 cells.

As an additional functional assessment of NMUR2 signaling, the agonists were tested in a beta‐arrestin recruitment and activity assay. Contrary to receptor‐mediated G_i_/cAMP or G_q_/calcium signaling, NY0116 and NY0128 did not recruit beta‐arrestin, even when the ligands were tested up to 10 μmol/L (Figure 2C). NY0128 also significantly decreased basal levels of beta‐arrestin recruitment and activity, whereas NY0116 did not (Figure 2D) (Table 2).

**Table 2:** It shows the EC50 and Emax (relative to NMS) values of NMS, NY0116, and NY0128 when evaluated in Gq mode. The EC50 and Emax values for NMS in a beta‐arrestin recruitment assay are also provided

	Calcium EC_50_ (nmol/L)	Calcium E_MAX_ (RFU)	b‐Arrestin EC_50_ (nmol/L)	b‐Arrestin E_MAX_ (% NMS)
NMS	ND	ND	1170 nmol/L ± 80	100%
NY0116	32 700 nmol/L ± 1100	2185 ± 205	NA	NA
NY0128	16 900 nmol/L ± 900	2036 ± 186	NA	NA

NMS, neuropeptide neuromedin S.

### 3.3 Pharmacokinetics study

**Figure 3:**
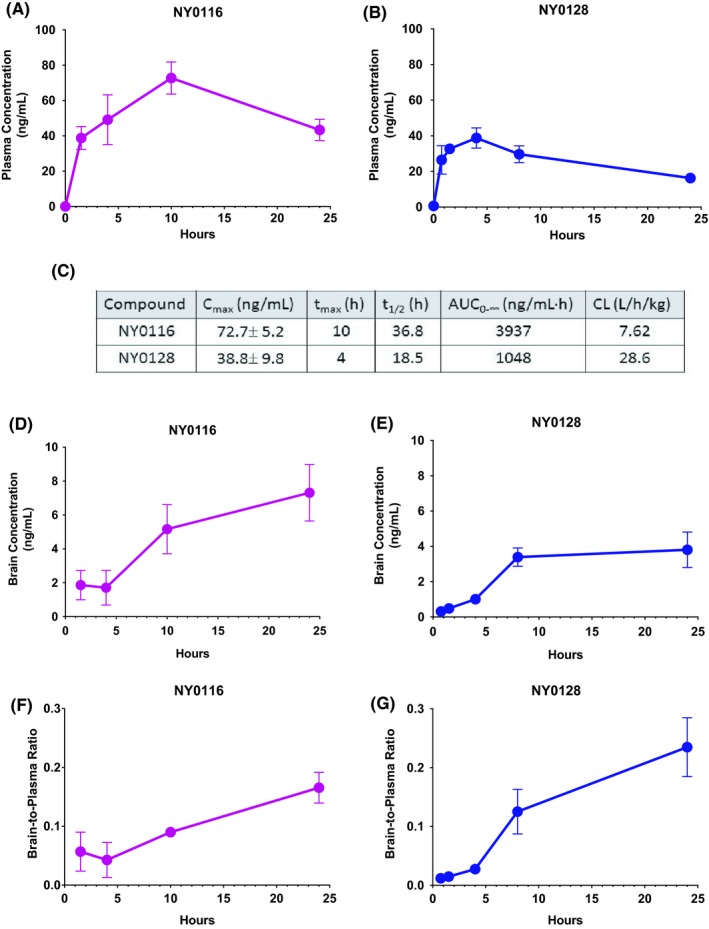
Pharmacokinetic analysis of rats’ blood plasma and brain concentrations of NMUR2 agonists after acute subcutaneous administration of compound NY0116 or NY0128 at 30 mg/kg. (A) Plasma concentrations for NY0116 were determined at 1.5, 4, 10, and 24 hours postinjection (*n *=* *3 for all time‐points, except at 10 hours when *n *=* *2). (B) Plasma concentrations for NY0128 were determined at 45 min, 1.5, 4, 8, and 24 hours postinjection (*n *=* *3 for all time points, except at 45 min when *n *=* *2). (C) Pharmacokinetic parameters for NY0116 and NY0128 determined by noncompartmental analysis. Pharmacokinetic analysis of (D) NY0116 and (E) NY0128 agonist concentrations in rats’ whole‐brain homogenates and the NY0116 (3F) and NY0128 (3G) brain‐to‐plasma ratio. Error bars represent standard error of the mean. NMUR1/2, NMU receptor ½

Plasma concentrations for NY0116 peak at 10 hours (*n *=* *3 for all time points, except at 10 hours when *n *=* *2) (Figure 3A). Plasma concentrations for NY0128 peak at 4 hours postinjection (*n *=* *3 for all time points, except at 45 min when *n *=* *2) (Figure 3B). According to Lipinski's “Rule of Five” for drug‐like properties,^25^ both compounds NY0116 and NY0128 have suitable physicochemical parameters (eg, cLogP = 3.37, tPSA = 71.13 for NY0116, and cLogP = 3.67, tPSA = 71.13 for NY0128 calculated by ChemBioDraw). Pharmacokinetic parameters for NY0116 and NY0128 determined by noncompartmental analysis (Figure 3C). Pharmacokinetic analysis of NY0116 (Figure 3D) and NY0128 (Figure 3E) concentrations in rats’ whole‐brain homogenates and the brain‐to‐plasma ratio of NY0116 (Figure 3F) and NY0128 (Figure 3G) shows that the compounds accumulate in the brain over time.

### 3.4 Acute NY0116 and NY0128 decrease food intake

**Figure 4:**
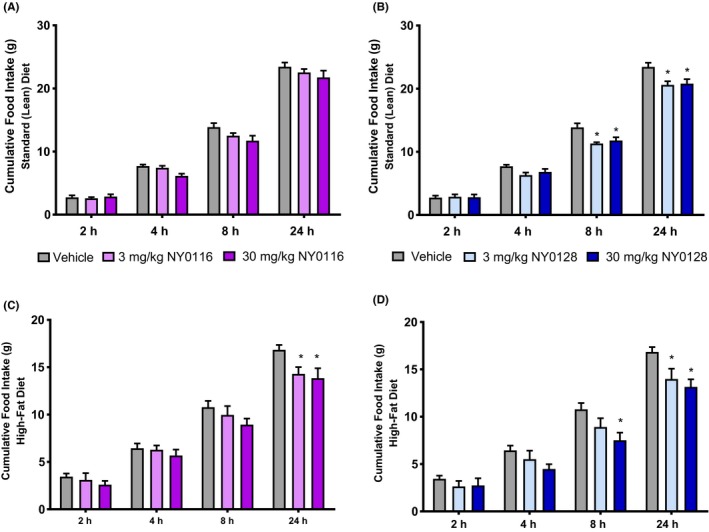
Acute NMUR2 agonists decrease food intake. (A) NY0116 did not significantly reduce 24‐hour standard diet intake at either dose (3 or 30 mg/kg, *n *=* *8 for both groups). (B) NY0128 at both doses (3 mg/kg, *n *=* *8; 30 mg/kg, *n *=* *6) significantly suppressed standard diet intake over 24 hours compared to vehicle (*n *=* *10). (C) NY0116 at both doses (9 or 90 mg/kg, *n *=* *6 for each group) significantly suppressed high‐fat diet intake compared to vehicle (*n *=* *10). (D) NY0128 at both doses (3 or 30 mg/kg, *n *=* *6) also significantly suppressed high‐fat diet intake compared to vehicle. Error bars represent standard error of the mean. *P <* *0.05 vs vehicle time‐point (repeated measures anova followed by Bonferroni posttest). NMUR1/2, NMU receptor ½

We evaluated the effects of NY0116 and NY0128 on intake of a standard diet and a high‐fat diet. NY0116 had no effect on consumption of a standard diet (main effect of drug treatment F(2,21) = 2.72, *P *=* *.09, effect of time F(3,63) = 1279, *P *<* *.001, and interaction F(6,63) = 1.67, *P *=* *.14) (Figure 4A). NY0116, however, inhibited intake of a high‐fat diet (main effect of drug treatment F(2,19) = 2.47, *P *=* *.11, effect of time F(3,57) = 1279, *P *<* *.001, and interaction F(6,57) = 2.86, *P *=* *.016) over 24 hours vs vehicle controls (Figure 4B). Post hoc analysis revealed that this effect was not significant at the 2, 4, and 8 hour time points, but was significant at both 9 and 90 mg/kg after 24 hours of cumulative food intake.

For NY0128, a significant reduction in standard diet consumption was observed (main effect of drug treatment F(2,22) = 8.64, *P *=* *.002, effect of time F(3,66) = 1007, *P *<* *.001, and interaction F(6,66) = 3.20, *P *=* *.008), an effect which was pronounced in both the 3 and 30 mg/kg treatments at the 8‐hour and 24‐hour time points (Figure 4C). NY0128 also suppressed intake of a high‐fat diet in a time‐dependent manner (main effect of drug treatment F(2,19) = 4.46, *P *=* *.026, effect of time F(3,57) = 599, *P *<* *.001, and interaction F(6,57) = 4.57, *P *<* *.001). Post hoc analysis indicates significant decreases at the 8‐hour time point (30 mg/kg only) and the 24‐hour time point (both doses) when compared with vehicle controls (Figure 4D).

### 3.5 Repeated NY0116 and NY0128 decrease bodyweight, VAT, and cholesterol on a high‐fat diet

**Figure 5:**
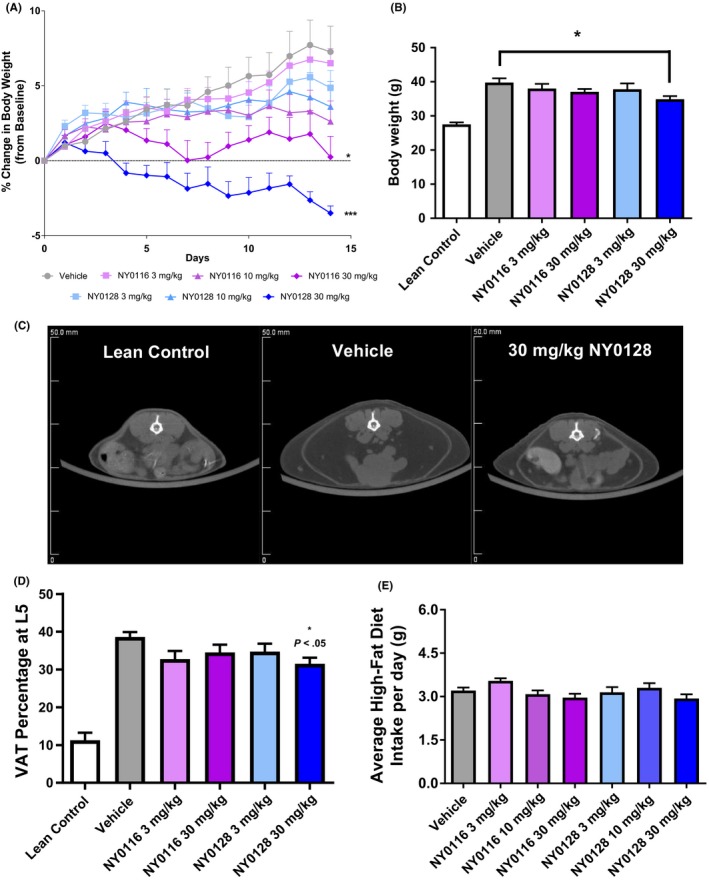
Repeat administration of NMUR2 agonists decrease body weight and visceral adipose tissue. (A‐B) Mouse body weight decreased in a dose‐dependent manner with administration of NMUR2 agonists. (C) Representative images of micro‐CT data at the L5 vertebrae displaying visceral adipose tissue (white arrow). (D) NY0128 at 30 mg/kg decreased percentage of visceral adipose tissue at L5, compared to vehicle (*n *=* *8–10 for each group). (E) Mouse food intake was not significantly changed by repeated administration of the NMUR2 agonists. Error bars represent standard error of the mean. *P <* *0.05 vs vehicle ***P <* *0.001 vs vehicle (A) repeated measures anova with Dunnett's posttest, (B–D) one‐way anova followed by Dunnett's posttest). NMUR1/2, NMU receptor ½

Compounds were administered over 14 days at doses of 3, 10, and 30 mg/kg. Both NY0116 and NY0128 treatments significantly decreased bodyweight (F(5,42) = 10.69, *P *<* *.001) compared to vehicle, whereas mice were maintained on a high‐fat diet (Figure 5A–B). To compare the amounts of VAT between the groups,^23^ we examined the cross section at the L5 vertebrae, using Inveon Research Workplace for quantification. Representative images of the cross sections quantified are shown in Figure 5C. There was a significant main effect (F(5,44) = 26.86, *P *<* *.001) and the high dose of NY0128 significantly decreased VAT percentage compared to vehicle (Figure 5D). Food intake was measured daily with no significant effect seen during the study (Figure 5E). Serum chemistry analysis showed a significant decrease (F(7,45) = 32.48, *P *<* *.001) in cholesterol (Figure S1) at the highest dose of NY0128, with no change in blood urea nitrogen (BUN) (Figure S1). There were also no observed drug‐induced changes in creatinine and aspartate aminotransferase (AST) (Figure S1).

## 4 DISCUSSION

In this study, we evaluated the potency and efficacy of two small molecule NMUR2 agonists in vitro and in vivo, previously only evaluated in an in vitro model measuring calcium accumulation. NMUR2 has been shown to signal through both the G_i_ and G_q_ G‐protein signaling pathways.^11^ Our study extends our knowledge of NMUR2 agonists activity beyond that of G_q_‐calcium,^21^ to include modulation of cAMP signaling. All three agonists, NMU‐8, NY0116, and NY0128, showed a dose‐dependent decrease in cAMP. These decreases in cAMP are likely due to G_i_ coupling of NMUR2, as the agonist action was sensitive to the known G_i_ inhibitor, PTX.^26^ The compounds exhibited low nanomolar potency when inhibiting cAMP and are more efficacious than NMU‐8, with NY0128 eliciting almost twice the effect elicited by NMU‐8. These data suggest they are acting as superagonists^27^ because their effects are greater than the effect of the endogenous agonist NMU‐8 at the NMUR2 through the G_i/o_ signaling pathway.

When evaluated for their ability to signal through the G_q_‐calcium signaling pathway, the compounds were far less potent, with EC50 values in the micromolar range. When tested in HEK293 cells that do not express NMUR2, neither compound showed a change in calcium at any dose, verifying the calcium signaling activity is due to NMUR2. A third possible NMUR2 signaling mechanism of the ligands is via the beta‐arrestin pathway and in theory, the ligands might signal via this pathway independently of G proteins. Contrary to receptor‐mediated activation of G_i_ or G_q_ signaling pathways, NY0116 and NY0128 surprisingly did not recruit beta‐arrestin. NY0128 at 10 μmol/L displayed a significant inverse agonism, further indicating the ligands are not NMUR2 agonists via beta‐arrestins. Since beta‐arrestin recruitment often promotes receptor desensitization resulting in decreased G protein signaling,^28^ this general lack of beta‐arrestin activity by NY0128 and NY0116 could be an important factor supporting sustained agonist activity at NMUR2 to ultimately drive weight‐loss during prolonged drug treatments. Taken together, these results suggest NY0116 and NY0128 are NMUR2 agonists for G_i_‐mediated cAMP inhibition and G_q_‐mediated calcium signaling and have undetectable and/or weak potency for beta‐arrestin recruitment.

In order to understand the pharmacological properties of the compounds for future studies, initial pharmacokinetic studies were conducted, with rats receiving NY0116 or NY0128 (30 mg/kg). Both compounds had appreciable plasma concentration values, with NY0116 reaching the highest concentration in both the plasma and the brain. NY0116 also reached maximum plasma concentration twice as fast as NY0128, while also having a half‐life twice as long. Although plasma levels of NY0128 stayed lower than NY0116, NY0128 had an increased brain‐to‐plasma ratio, with compound accumulating in the brain up to 10 hours post administration. The brain exposure is critical to the work conducted in this paper, because we are targeting a centrally expressed receptor,^13^, ^29^ therefore it is crucial that our compounds cross the blood brain barrier. The presence of the compounds in the brain suggests the compounds have access to the intended target, NMUR2. The concentrations in the brain homogenate are comparable to the EC50s from the cell assays. Furthermore, the timing‐course of brain access and inhibition of food intake occur at the same time, thus providing evidence that the compounds are acting on NMUR2 in the brain. This is particularly important when using these tool compounds as scaffolds to design other small molecule NMUR2 agonists. The ability to penetrate the blood brain barrier, the potency, and the efficacy of these compounds provides us with an advantageous starting point in developing a pharmacotherapy targeting NMUR2.

Previous studies have shown that NMUR2 plays an important role in feeding, with one showing central administration of NMU significantly decreased food intake^20^ leading us to investigate the effect of the compounds on food intake. A single dose of compound significantly decreased consumption of high‐fat diet, 24 hours after treatment. NY0128 also showed a decrease at the 8 hour time point of high‐fat diet consumption, as well as at the 8 and 24 hour time points of standard diet consumption. These data suggest that both compounds affect food intake similar to NMU.

Lastly, the compounds showed a decrease in adiposity after chronic subcutaneous treatment. Compound was administered once per day for 14 days. Excitingly, the high dose of NY0128 not only prevented weight gain but also resulted in weight loss while also reducing cholesterol levels compared to vehicle. This is complemented by the NY0128 30 mg/kg reduction in VAT and together suggests that part of the observed weight reduction with NY0128 is due to decreased VAT. The highly efficacious results seen with NY0128 in vivo further compliments our in vitro data, where NY0128 is the more potent and efficacious compound. The concentrations found effective in vitro are similar to the brain exposure of NY0128. This further suggests that NY0128 is acting on the centrally expressed NMUR2 to promote fat loss and decrease food intake. Taken together, these data suggest that NY0116 and NY0128 are promising tool compounds in targeting NMUR2 as a pharmacotherapy for obesity.

## 5 CONCLUSION

We provide strong evidence that two small‐molecule agonists for NMUR2 activate both G_i_‐mediated cAMP inhibition and G_q_ ‐mediated calcium signaling, achieve appreciable brain levels after a single subcutaneous dose, and decrease high‐fat diet intake in rats. Although NY0116 and NY0128 are not selective for NMUR2 (also act on NMUR1), our laboratory and others have demonstrated that NMUR2 in particular is a key regulator of feeding behavior,^7^, ^19^, ^30^ and therefore the likely target for these agonists. Furthermore, despite being fed a high‐fat diet, repeated administration of NY0128 reduces body weight, VAT, and cholesterol in obese mice. Although the NMUR2 agonists presented here require further optimization, these small molecules demonstrate promising effects as lead drug candidates on feeding, fat content and body weight composition. Taken together, this work indicates NMUR2 agonism has pharmacotherapeutic potential for addressing metabolic disorders, such as obesity.

## AUTHOR CONTRIBUTIONS

Participated in research design: Hommel, Allen, Zhou. Conducted experiments: Sampson, Kasper, Felsing, Raval, and Ye. Performed data analysis*:* Sampson, Kasper, Felsing, Raval, Patrikeev, and Rytting. Wrote or contributed to the writing of the manuscript: Sampson, Kasper, Allen, and Hommel.

## Supporting information


**Figure S1.** Repeat administration of NMUR2 agonists significantly lower cholesterol. (A) Mouse cholesterol levels decreased with repeated administration of NY0128 at 30 mg/kg. (B–D) Repeat administration of NMUR2 agonists had no effect on creatinine, AST, and ALT. Error bars represent standard error of the mean. *P <* *.05 vs vehicle ***P <* *.001 vs vehicle. One‐way anova followed by Dunnett's posttest was used.
