# Supplementary material for: Small‐Molecule Neuromedin U Receptor 2 Agonists Suppress Food Intake and Decrease Visceral Fat in Animal Models
Source: Pharmacol Res Perspect. 2018 Aug 23;6(5):e00425. doi: 10.1002/prp2.425 (PMC6106167; doi:10.1002/prp2.425)
Supplement: Supplementary file 1 — Figure S1. Repeat administration of NMUR2 agonists significantly lower cholesterol. (A) Mouse cholesterol levels decreased with repeated administration of NY0128 at 30 mg/kg. (B–D) Repeat administration of NMUR2 agonists had no effect on creatinine, AST, and ALT. Error bars represent standard error of the mean. *P < .05 vs vehicle ***P < .001 vs vehicle. One‐way anova followed by Dunnett's posttest was used. [file PRP2-6-e00425-s001.docx]

**Supplemental Figure 1.** Repeat administration of NMUR2 agonists significantly lower cholesterol. (A) Mouse cholesterol levels decreased with repeated administration of NY0128 at 30mg/kg. (B,C,D) Repeat administration of NMUR2 agonists had no effect on creatinine, AST, and ALT. Error bars represent standard error of the mean. * *p*<.05 vs vehicle *** *p*<.001 vs vehicle. One-way ANOVA followed by Dunnett’s post-test was used.
